# Platelet storage induces accelerated desialylation of platelets and increases hepatic thrombopoietin production

**DOI:** 10.1186/s12967-018-1576-6

**Published:** 2018-07-18

**Authors:** Jooyoung Cho, Hyunkyung Kim, Jaewoo Song, June-Won Cheong, Jeong Won Shin, Woo Ick Yang, Hyun Ok Kim

**Affiliations:** 1 Department of Laboratory Medicine, Yonsei University College of Medicine, 50-1 Yonsei-ro, Seodaemun-gu, Seoul, 03722 South Korea; 2 Department of Laboratory Medicine, Yonsei University Wonju College of Medicine, Wonju, South Korea; 3 Department of Obstetrics and Gynecology, Gangnam Severance Hospital, Yonsei University College of Medicine, Seoul, South Korea; 4 Department of Internal Medicine, Yonsei University College of Medicine, Seoul, South Korea; 5 Department of Laboratory Medicine, Soonchunhyang University School of Medicine, Seoul, South Korea; 6 Department of Pathology, Yonsei University College of Medicine, Seoul, South Korea

**Keywords:** Platelet storage lesion (PSL), Desialylation, Thrombopoietin (TPO), Ashwell–Morell receptor (AMR)

## Abstract

**Background:**

Stored platelets undergo deleterious changes, referred to as platelet storage lesions (PSLs), which accelerate the desialylation of platelets and result in their phagocytosis and clearance by hepatic macrophages. Recent studies have reported that Ashwell–Morell receptor binds to desialylated platelets, thereby inducing hepatic thrombopoietin (TPO) production in a mouse model. Therefore, this study aimed to demonstrate these relationships between PSL and hepatic TPO production in human study.

**Methods:**

Platelet concentrates (PCs) were obtained from 5 healthy volunteers and the remaining were discarded samples from the blood bank. PCs were divided into two halves, and stored either at 22 or 4 °C. Experiments were conducted using serial samples. Desialylation was assessed using flow cytometry, and structural changes were visualized using electron microscopy. Following co-culture of HepG2 cells (HB-8065, ATCC) with isolated platelets, hepatic TPO production was determined using real-time quantitative polymerase chain reaction and the supernatant TPO level was measured using a Luminex kit.

**Results:**

For 5 days of storage duration, platelet counts were not influenced by the storage conditions, but the degree of desialylation was proportional to the storage duration. Significant changes in the platelet surface and structure according to storage conditions were noted in electron microscopy. HepG2 cells incubated with aged platelets expressed more *TPO* mRNA, and supernatant TPO levels were proportional to the storage duration. Refrigeration also influenced on the results of this study, but they were not statistically significant.

**Conclusions:**

This is the first study to demonstrate that, in vitro, aging and refrigeration affect the integrity of human platelets, resulting in induction of hepatic TPO mRNA and protein expression.

## Background

The transfusion of platelet components is widely used as a life-saving therapy to stop bleeding and maintain stable hemostatic status [1, 2]. Platelet components are routinely provided in the form of platelet concentrates (PCs) derived from donated whole blood or plateletpheresis, and can be stored for up to 5 days at room temperature (22 ± 2 °C) with continuous gentle agitation [3, 4]. Longer platelet storage duration has not been recommended because of the possible risk of bacterial contamination [5, 6], and as a result, blood transfusion services are faced with a chronic shortage of platelets [3]. Although new methods of pathogen inactivation are being developed for application in clinical settings [7], questions have been raised about whether a storage duration of longer than 5 days is helpful for blood banks, considering the many existing risks [2, 6, 8]. Refrigeration may lower the risk of bacterial contamination; however, investigators have noted that hypothermic conditions (temperatures lower than 15 °C) can have deleterious structural and functional effects on platelets [3, 5, 9, 10]. For these reasons, the storage duration of PCs is currently limited to 5 days.

In addition to the possibility of bacterial contamination, platelet aging can affect their structural and functional integrity [2, 4, 6, 11, 12]. Platelets in PCs, which are collected in gas-permeable plastic bags and used routinely in blood banks, undergo many physiologic changes during collection, processing, and storage. These changes can alter the structure and function of platelets and are referred to as the platelet storage lesion (PSL) [2, 6, 12]. The PSL may, in part, be due to desialylation. Platelets secrete sialidase under many circumstances, such as the cooling–rewarming process, aging, and other pathologic statuses [1, 8]. Sialidase induces hydrolysis of sialic acid from glycoprotein (GP) 1bα or GPV on the platelet surface, forming irreversible clustering of the von Willebrand factor (vWF) receptor [6, 8, 13]. Moreover, Hoffmeister et al. demonstrated that the recognition of β-*N*-acetylglucosamine (β-GlcNac) residues on clustered GPIbα by the αMβ2 integrin receptor on hepatic macrophages (Kupffer cells) produced in vivo platelet phagocytosis in mice [5]. PCs stored at hypothermic condition can be more easily altered than those stored at room temperature, and as a result, more rapid desialylation and clearance can occur [3, 8, 11, 12].

Thrombopoietin (TPO) is the main regulator of megakaryopoiesis and thrombopoiesis [14–17]. It stimulates platelet production by promoting differentiation of hematopoietic stem cells (HSCs) into the megakaryocytic lineage [15, 16]. This cytokine is predominantly produced in the liver at a constant rate, but its level is regulated by circulating platelets. Platelets and megakaryocytic cells express the TPO receptor, known as the myeloproliferative leukemia oncogene (Mpl) receptor, which binds and internalizes TPO in thrombocytosis [14, 15]. In thrombocytopenia, low levels of Mpl receptor expression and TPO clearance facilitate an increase of thrombopoiesis. Folman et al. demonstrated that the plasma TPO level is inversely proportionate to circulating platelet counts, and that the Mpl receptor plays a major role in TPO clearance and platelet homeostasis [14].

Aged, refrigerated, or damaged platelets undergo many physiologic changes, such as PSL [2, 6, 12]. These altered platelets are more susceptible to phagocytosis by hepatic macrophages than normal platelets [3]. In addition, recent studies noted that aged platelets stimulate hepatic TPO production [1, 18, 19]. Grozovsky et al. demonstrated that desialylated platelets are cleared by the hepatic endocytic Ashwell–Morell receptor (AMR) and thereby stimulate hepatic TPO production by the JAK2-STAT3 pathway [1]. Another study suggested that regardless of the plasma platelet count, the rate of hepatic TPO production is constant [15].

This study aimed to investigate the relationship between desialylation of platelets and hepatic TPO production. We hypothesized that aged or refrigerated PCs are cleared more rapidly than control PCs by hepatic endocytic AMR, and thereby hepatic TPO production would be increased. Therefore, this study investigated changes in platelets during storage, in order to develop guidelines concerning appropriate storage conditions for platelet components.

## Methods

### Samples and subjects

Five healthy volunteers, who agreed to participate in this study and signed the informed consent, were enrolled. Three men and two women participated, and their ages ranged from 26 to 58 years. All participants were free of hematologic and oncologic diseases and were not taking medications that could affect platelet function. They visited the blood bank in Severance Hospital (Seoul, Korea) once for the purpose of blood donation from March to August 2016.

The median vein near the antecubital fossa was punctured with an 18-gauge needle attached to blood collecting set. The average time for collecting 400 mL of whole blood (WB) sample was less than 10 min. Each sample of WB was drawn into a triple blood bag (Triple BSDC-NP-SB3; Green Cross Medical Science Corp., Yongin, Korea) containing 56 mL of citrate phosphate dextrose adenine (CPDA)-1. Component processing was performed using centrifugation with a Kubota 8730 (Kubota corp., Osaka, Japan) at 2000 rpm for 5 min in order to separate red blood cells (RBCs). This was followed by centrifugation at 4000 rpm for 6 min in order to separate the plasma component. Approximately 50 mL of PC was obtained from each WB sample during the blood processing procedure, and each PC was transferred to a gas-permeable transfer plastic bag (Green Cross Medical Science Corp.) for analysis. Besides the 5 donated PCs, some PCs that were being discarded by the blood bank were also used under the authorization of the institutional review board (IRB).

To study the effects of refrigeration on platelets, each PC was subdivided into two groups and stored either at 4 °C or at 22 °C. Samples from serially stored platelets were examined at 0 (fresh), 1, 3, and 5 days after donation. This study was approved by the IRB of Severance Hospital (No. 4-2015-0105).

### Platelet preparation

Platelet counts of each group were determined using an ADVIA 2120i (Siemens Diagnostics, Tarrytown, NY, USA) automated complete blood cell (CBC) analyzer. In order to prepare for flow cytometry, a washing procedure was performed as described by Jansen et al. [8]. Platelets were washed with a buffer composed of 140 mM NaCl, 5 mM KCl, 12 mM trisodium citrate, 10 mM glucose, 12.5 mM sucrose, and 1 µg/mL prostaglandin E_1_ with a pH of 6.0 (buffer A; washing buffer). They were then resuspended in 10 mM HEPES, 140 mM NaCl, 3 mM KCl, 0.5 mM MgCl_2_, 10 mM glucose, and 0.5 mM NaHCO_3_ with a pH of 7.4 (buffer B; suspension buffer) [1, 8]. Platelets treated with α2-3,6,8 neuraminidase from *Clostridium perfringens* (sialidase) (Sigma-Aldrich, Saint Louis, MO, USA) to remove surface sialic acid were used as a positive control. Isolated platelets (1 × 10^8^) from each group were mixed with 1 mL of buffer A, followed by centrifugation using an Eppendorf Centrifuge 5424 (Eppendorf Inc., Hamburg, Germany). After removal of the supernatant, platelet pellets were resuspended in 1 mL of buffer B and were subdivided into two microtubes for performing assays on different kinds of lectins. All of these procedures were conducted at room temperature (22 °C).

### Flow cytometry

Desialylation of platelets was confirmed by lectin binding, using flow cytometric analysis. Fluorescein isothiocyanate (FITC)-conjugated *Ricinus Communis* agglutinin I (RCA-I; Vector Laboratories, Burlingame, CA, USA) was used for assessing platelet surface β-GlcNac exposure [8, 13]. RCA-I at 5.0 µg/mL was added to each of isolated platelets after resuspension in buffer B. In the positive control group, 0.3 U/mL of sialidase was also added. The groups of platelets stored at 4 °C were then incubated at 37 °C for 20 min, using a WiseTherm^®^ HB-R (Daihan Sci., Seoul, Korea) water bath, and the groups of platelets stored at 22 °C were incubated with continuous gentle agitation. The lectin binding of platelets was analyzed using a Beckman Navios flow cytometer (Beckman Coulter Inc., Brea, CA, USA). Platelets were gated according to their forward scatter (FSC) and side scatter (SSC) characteristics.

### Electron microscopy

Structural changes in platelets were examined using scanning electron microscopy (SEM) and transmission electron microscopy (TEM). For SEM, platelets were fixed with 2% glutaraldehyde–paraformaldehyde in 0.1 M phosphate buffer (PB) at pH 7.4 for 6 h and washed twice for 30 min in 0.1 M PB. They were post-fixed with 1% OsO_4_ dissolved in 0.1 M PB for 2 h and dehydrated in an ascending gradual series (50–100%) of ethanol. Afterwards, they were infiltrated using isoamyl acetate and subjected to a Critical Point Dryer (HCP-2; Hitachi, Tokyo, Japan). They were coated with gold using ion sputter (IB-3, Eiko, Fukuoka, Japan) at 6 mA for 6 min. Secondary electron images of the surfaces were obtained using scanning electron microscopy (SU-8220 FE-SEM; Hitachi) at an acceleration voltage of 20 kV in the KBSI Seoul Western Center.

For TEM, after dehydration in ethanol, specimens were embedded using a Poly/Bed 812 kit (Polysciences Inc., Warrington, PA, USA). After pure fresh resin embedding, polymerization was conducted at 65 °C in an electron microscope oven (TD-700; Dosaka EM, Kyoto, Japan) for 24 h. Sections of approximately 200–250 nm thick were initially cut and stained with toluidine blue (T3260; Sigma-Aldrich) for light microscopy. Thin sections of 70 nm were double stained with 6% uranyl acetate for 20 min (Electron Microscopy Sciences, Catalog No. 22400; Hatfield, PA, USA) and lead citrate for 10 min (Thermo Fisher, Waltham, MA, USA) for contrast staining. The sections were cut using a Leica EM UC-7 (Leica Microsystems, Wetzlar, Germany) with a diamond knife (Diatome Ltd., Biel, Switzerland), and transferred on copper and nickel grids. All thin sections were examined using transmission electron microscopy (JEM-1011; JEOL Ltd., Tokyo, Japan) at an acceleration voltage of 80 kV in the Severance Biomedical Science Institute.

### Co-culture of HepG2 cells with isolated platelets

HepG2 cells (HB-8065, ATCC) were cultured in 75 cm^2^ cell culture flasks (SPL Life Sciences, Gyeonggi, Korea); cells were maintained in Dulbecco’s modified Eagle’s medium (DMEM)/low glucose (Gibco, Green Island, NY, USA) supplemented with 10% fetal bovine serum (FBS; Gibco) and 1% penicillin/streptomycin (P/S; Gibco) at 37 °C and 5% CO_2_. For subculture, HepG2 cells were washed first with Dulbecco’s phosphate buffered saline (PBS; Biowest, Nuaillé, France), and cell detachment was performed using 0.5% trypsin–EDTA (Invitrogen, Waltham, MA, USA), followed by resuspension in cell culture medium.

For the assays, 1 × 10^6^ HepG2 cells were seeded into 6-well dishes (Nunclon Delta Surface; Thermo Scientific, Waltham, MA, USA). HepG2 cells were then counted using a Neubauer-improved (0.0025 mm^2^, depth 0.1 mm; Paul Marienfeld GmbH & Co. KG, Lauda-Königshofen, Germany) hemocytometer. After overnight incubation at 37 °C and 5% CO_2_, cells were washed using PBS and resuspended in calcium-free DMEM supplemented with 2% FBS and 1% P/S, followed by incubation with 1 × 10^7^ isolated platelets for 6 h. Supernatants from each well were then collected in a 2 mL microtube, and the cells were detached using cell scrapers. All of the cells and supernatants were stored at − 20 °C until analysis.

### Measurement of TPO in supernatant of HepG2 cell culture

TPO concentration in the cell culture supernatant was measured using a Magnetic Luminex^®^ Performance Assay Human TPO kit (catalog No. LUHM288) (R&D Systems Inc., Minneapolis, MN, USA). This assay has a working range from 81 to 11,379 pg/mL. The intraassay coefficient of variation was < 5.6% and the interassay coefficient of variation was < 8.3%, according to the manufacturer.

### *TPO* mRNA expression

For the polymerase chain reaction (PCR) study, total RNA was extracted using an RNeasy^®^ Plus Mini kit (Qiagen, Hilden, Germany). The RNA concentration was measured using a Nanodrop Lite (Thermo Scientific) at an absorbance of 260/280 nm, and about 1 µg of extracted RNA was used for cDNA synthesis. First-strand cDNA was performed using oligo(dT)-primed reverse transcription with genetically engineered Moloney murine leukemia virus (MMLV) reverse transcriptase (SuperScript^®^ III First-Strand; Invitrogen). Synthesized cDNA was stored at − 20 °C for real-time quantitative PCR (qPCR). For real-time qPCR, commercially available *TPO* (Hs01061346_m1; Thermo Scientific) and *GAPDH* (Hs02758991_g1; Thermo Scientific) probes were used. Required cycles for PCR amplification were 27 cycles (*GAPDH*) and 35 cycles (*TPO*), and each cycle consisted of 30 s of denaturation at 94 °C, 30 s of annealing at 55 °C, and 1 min of extension at 72 °C. PCR was performed using a StepOnePlus Real Time PCR system (Applied Biosystems) and analyzed using StepOne Software version 2.3 (Applied Biosystems). Following real-time PCR, threshold cycle values (C_T_ values) were obtained on the amplification curves. All samples were measured in triplicate and the mean C_T_ value for each was used for quantitation. *TPO* mRNA quantitation was calculated using the E-ΔC_T_ formula, and *GAPDH* expression was used as a control.

### Statistical analysis

Numerical values were expressed as the mean ratio compared with that of the fresh controls (Day 0). Data were analyzed using a Student’s *t*-test (for 2 groups) or a one-way analysis of variance (ANOVA) (for more than 3 groups) followed by post hoc analysis. SPSS software (Version 18.0; IBM corp., Armonk, NY, USA) and Microsoft^®^ Excel software (Version 14.0; Microsoft corp., Redmond, WA, USA) were used for statistical analysis. A *P* value of less than 0.05 was considered statistically significant.

## Results

### Platelet counts according to storage conditions

Table 1 shows the mean platelet counts and mean platelet volumes at various storage durations and temperatures. Platelet counts determined using an automatic CBC analyzer were approximately 1.3–1.5 × 10^6^/µL for each of the groups. An analysis using Student’s unpaired *t*-test revealed there were no statistically significant differences between the 22 and 4 °C groups. Moreover, a one-way ANOVA followed by post hoc analysis showed there were no significant changes in platelet counts according to the time course (*P *> 0.05). No correlation was observed between storage conditions and platelet counts in vitro.

**Table 1:** Platelet counts in different storage durations and temperatures

	D + 0	D + 1	D + 3	D + 5	D + 7
22 °C					
Platelet count (× 10^3^/μL)	1383.3 ± 149.9	1375.7 ± 73.4	1334.7 ± 221.3	1312.7 ± 75.1	1401.3 ± 246.2
MPV (fL)	9.1 ± 0.2	9.3 ± 0.2	9.0 ± 0.4	9.4 ± 0.7	8.8 ± 0.9
4 °C					
Platelet count (× 10^3^/μL)		1363.3 ± 76.0	1302.7 ± 40.8	1246.3 ± 151.2	1428.0 ± 175.9
MPV (fL)		7.1 ± 0.8	7.0 ± 0.8	7.5 ± 0.5	7.2 ± 0.6

Data are expressed as mean ± standard deviation in each group (n = 5)

### Flow cytometric analysis of desialylation of platelets

**Fig. 1:**
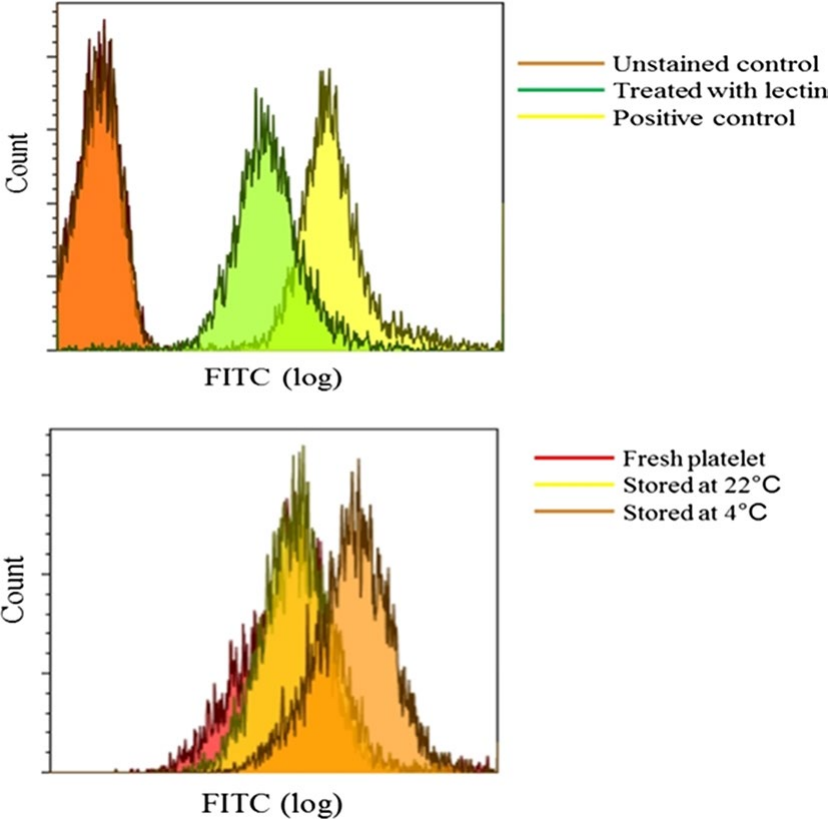
Desialylation of platelet surface in vitro. Desialylation was assessed by flow cytometry using FITC conjugated RCA-I lectin. **a** Human platelets stained with RCA-I lectin, unstained platelets (negative control) and platelets treated with α2-3,6,8 neuraminidase from *Clostridium perfringens* and stained with RCA-I lectin stained (positive control). **b** Comparison between platelets with different storage conditions: Day 0 (fresh), Day 1 (stored at 22 °C), and Day 1 (stored at 4 °C)

Human platelets show some desialylation as demonstrated by greater reactivity in a flow cytometry assay with FITC conjugated RCA-I lectin than unstained control platelets. Furthermore, human platelets treated with sialidase (positive control) showed more desialylation than untreated platelets (Fig. 1a). Both storage duration and temperature affected platelet desialylation. Compared with fresh (Day 0) platelets, those stored for 1 day showed increased lectin binding and platelets stored at 4 °C showed more positivity than those stored at 22 °C (Fig. 1b).

**Fig. 2:**
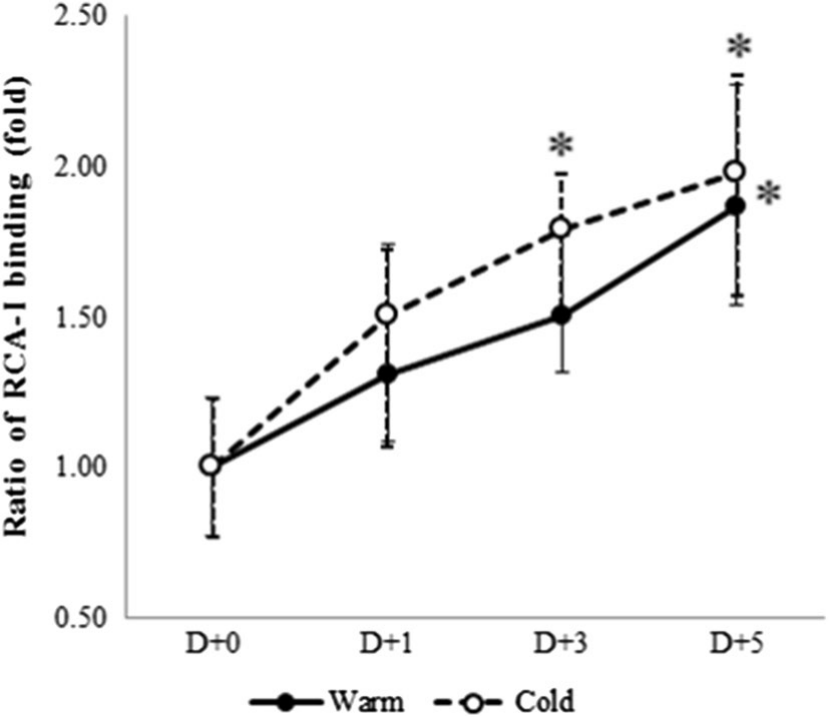
Flow cytometric analysis of β-GlcNac exposure on platelets using FTIC-conjugated RCA-I lectin. Arithmetic mean values of FITC (log) were used for evaluation. Values in these graphs represent the relative ratio to fresh platelets. RCA-I lectin was used for detection using flow cytometry. Closed circles represent the platelets stored at 22 °C, open circles represent platelets stored at 4 °C (n = 4). *P < 0.05 represents statistical significance

Platelet surface exposure of β-GlcNac as assessed by flow cytometry using RCA-1 lectin showed a positive correlation with storage duration. The level of desialylation was expressed as the mean relative ratio of samples compared with the mean of fresh controls. As expected, β-GlcNac exposure on the surface of platelets increased depending on the storage duration and was statistically significant on Day 5 of platelets stored at 22 °C and from Day 3 to Day 5 of platelets stored at 4 °C (*P *< 0.05). Refrigeration also influenced desialylation of platelets, but the differences between platelets stored at 22 °C and those stored at 4 °C were not statistically significant (*P *> 0.05) (Fig. 2). Positive controls (groups treated with sialidase) demonstrated the greatest ratio of RCA-I lectin binding (data not shown).

### Structural changes of aged or refrigerated platelets in vitro

**Fig. 3:**
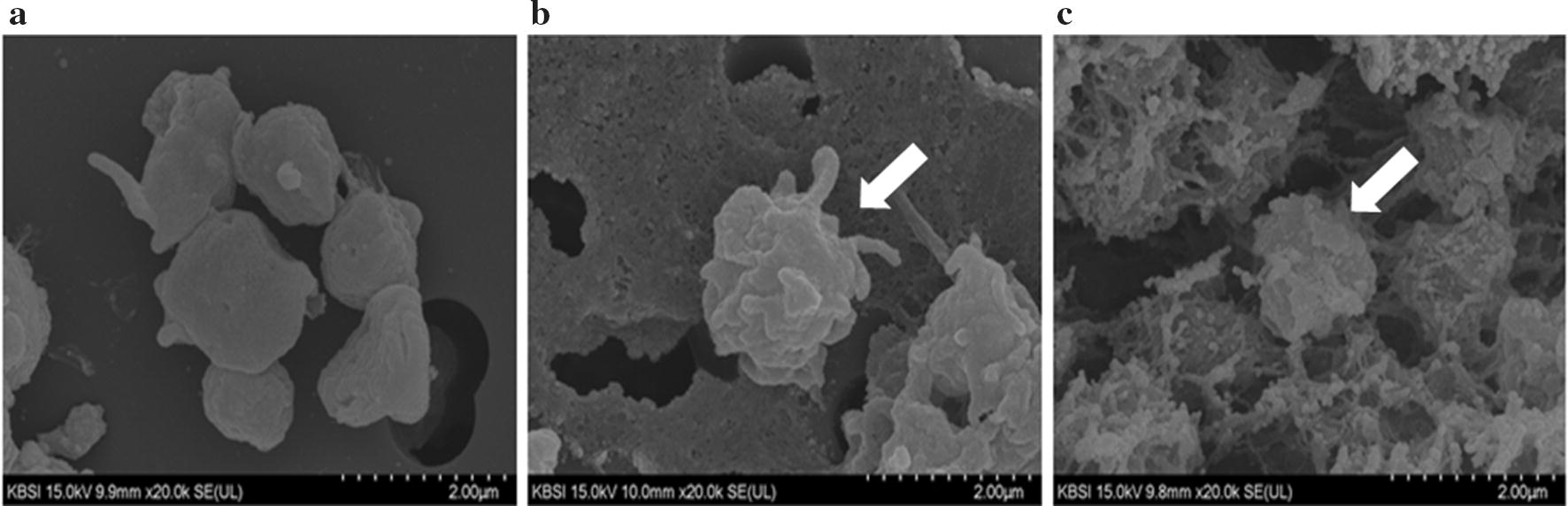
Scanning electron microscopy (SEM) of the platelets at different storage durations and temperatures. Aged and refrigerated platelets showed morphologic changes including bulbous protrusions and pseudopods, indicating platelet activation. Magnification, ×20,000: **a** fresh platelets, **b** platelets stored at 22 °C for 2 days, and **c** platelets stored at 4 °C for 2 days

Electron microscopic visualization was conducted using both SEM and TEM. At 20,000× magnification, SEM analysis showed that platelets stored at 4 °C display morphologic change from discoid to spheroid with a number of bulbous protrusions and pseudopods with, indicating platelet activation [20]. And platelets stored at 22 °C for 2 days showed more changes than fresh platelets, but less changes than platelets stored at 4 °C. It is known that refrigeration accelerates activation with rearrangement of surface configuration of platelet surface receptors (Fig. 3) [5].

**Fig. 4:**
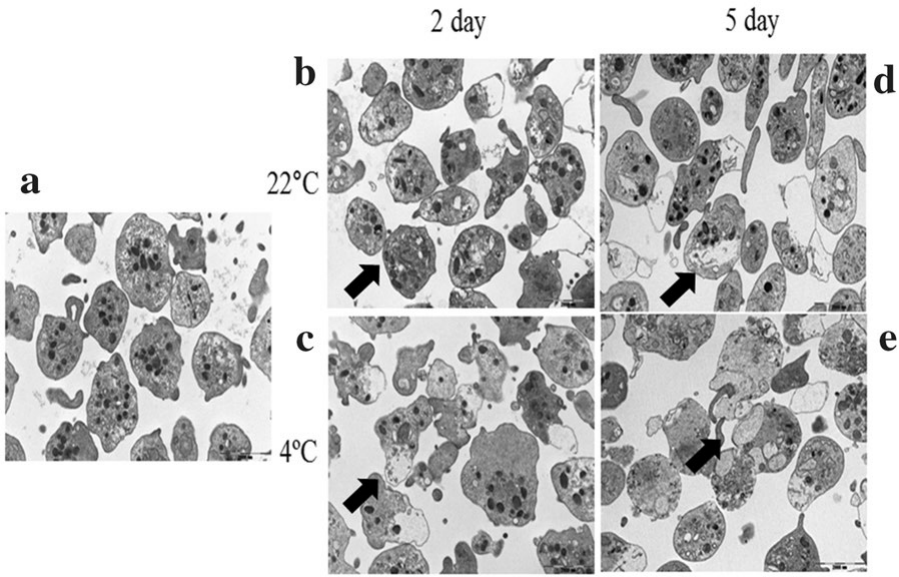
Transmission electron microscopy (TEM) of the platelets at different storage durations and temperatures. Magnification, ×12,000: fresh platelets (**a**), platelets stored at 22 °C for 2 days (**b**), platelets stored at 4 °C for 2 days (**c**), platelets stored at 22 °C for 5 days (**d**), and platelets stored at 4 °C for 5 days (**e**). Aged platelets showed morphologic changes including changes in alpha granule and cell shape (**d**, **e**), and refrigerated platelets showed damaged cell membrane resulting in release of granules and cell organelles (**c**, **e**)

In TEM analysis at 12,000× magnification, longer storage accelerated platelet activation, showing morphologic changes including elongation of projections, condensation and fusion of alpha granules, and changes in cell shape. Furthermore, platelets stored at 4 °C display damage and protrusion of cell membrane, resulting in release of granules and cell organelles (Fig. 4) [21]. Both a longer storage duration and refrigeration activated and influenced the shape and structure of the platelet surface.

### Supernatant TPO levels of HepG2 cell cultures

**Fig. 5:**
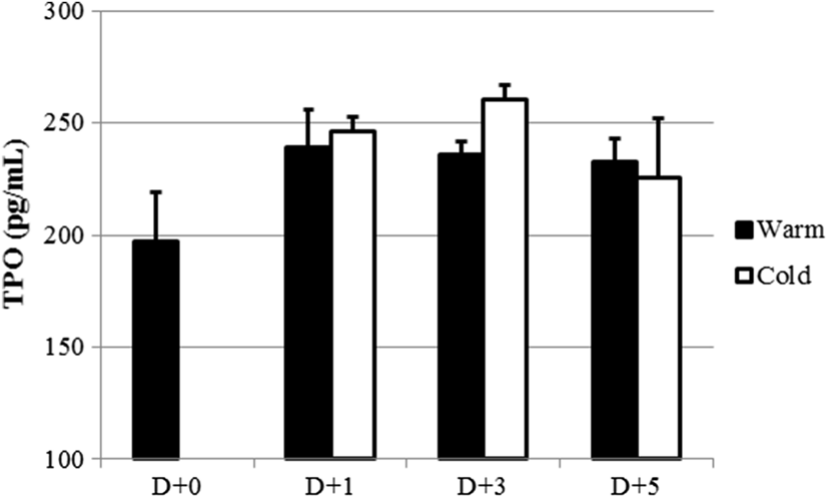
TPO levels in supernatant of HepG2 cell cultures after incubation with human platelets. After incubation with human platelets for 6 h under various conditions, supernatant TPO levels were measured using a Magnetic Luminex^®^ Performance Assay Human TPO kit. Closed bars represent the platelets stored at 22 °C and open bars represent the platelets stored at 4 °C (n = 3). There were no significant changes in supernatant TPO levels according to time course and storage temperature (*P *> 0.05)

As mentioned above, it was hypothesized that as platelets stored for a longer duration were incubated with HepG2 cells, supernatant TPO levels would increase. TPO levels in supernatant of HepG2 cell culture increased from Day 0 to Day 3. In supernatant of HepG2 cell cultures that were reacted with platelets stored at 4 °C, TPO levels were higher than in that of HepG2 cell cultures that reacted with platelets stored at 22 °C (Fig. 5).

### Hepatic *TPO* mRNA expression

**Fig. 6:**
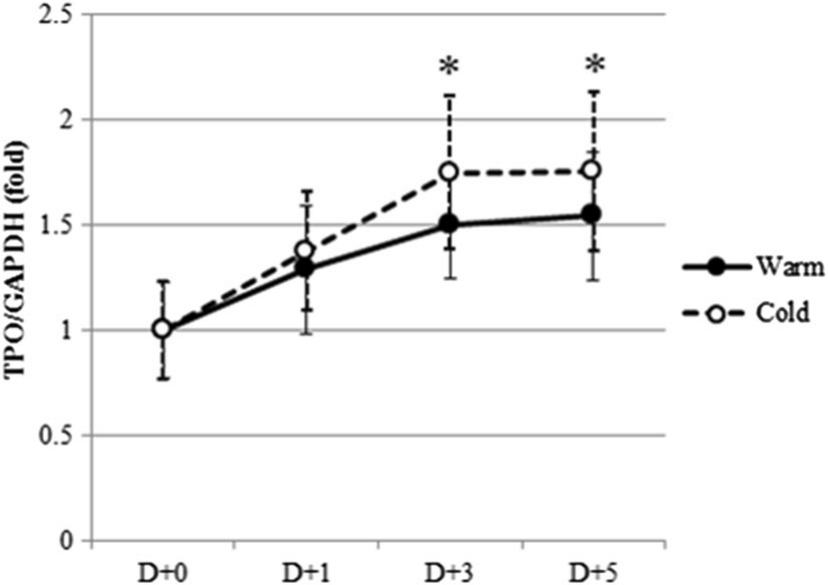
Quantitation of hepatic *TPO* mRNA expression using real-time qPCR. Values in these graphs represent the relative ratio to fresh (D + 0) platelets. 1 × 10^6^ HepG2 cells were incubated with 1 × 10^7^ isolated platelets for 6 h. Closed circles represent the platelets stored at 22 °C and open circles represent the platelets stored at 4 °C (n = 5). *P < 0.05 represent statistical significance

HepG2 cells treated with isolated human platelets expressed *TPO* mRNA as measured using real-time qPCR. Figure 6 shows the relative ratio of *TPO/GAPDH* compared with the mean ratio of fresh controls. Groups of platelets assessed after 6 h of incubation showed more significant changes than those measured after 24 h of incubation (data not shown). The relative ratio of *TPO/GAPDH* mRNA expression increased according to storage duration and refrigeration (*P *< 0.05 for Day 3 and 5 of platelets stored at 4 °C, but not for platelets stored at 22 °C).

## Discussion

A timely and adequate supply of PCs is essential for many blood banks and healthcare facilities. However, a chronic shortage of PCs remains a problem [3]. Since the 1960s, PCs have been stored at room temperature with gentle agitation, but their storage duration has been limited to 5 days [4, 6, 8]. Technical developments for longer storage of PCs have been established; however, extension of the storage duration is restricted in routine blood banks because of the possible risk of bacterial contamination [5, 6]. It is known that hypothermal conditions alter the structural and functional characteristics of platelets and results in what is referred to as PSL [2, 6, 12]. It is also known that damaged platelets are easily cleared from the circulation by phagocytosis of hepatic Kupffer cells [3, 5, 8].

As mentioned above, Grozovsky et al. identified that aged platelets are recognized by hepatic endocytic AMR, and this is followed by stimulation of hepatic *TPO* mRNA expression via the JAK2-STAT3 pathway [1]. Aging and refrigeration also accelerate desialylation of platelet surface. Therefore, it was hypothesized that hepatic cell uptake of aged or refrigerated platelets triggers hepatic TPO production. This study investigated human platelet desialylation and hepatic *TPO* mRNA expression to establish the relationship between storage conditions (such as storage duration and temperature) and changes in platelets in routine blood banks. The data presented in this study supported the hypothesis concerning these relationships.

First, stored platelet counts were not influenced by storage conditions such as storage duration or refrigeration. During storage, platelets in PCs undergo a number of changes including activation, clustering of surface receptors and morphologic changes. In this study, platelet counts were not significantly reduced due to platelet clumping, adhesion to plastic bag, or even cell death during storage even in 4 °C [22], indicating that increased TPO level is not due to decreased platelet counts.

Second, platelet surface β-GlcNac exposure was proportional to the storage duration and temperature. Positive platelet controls that were treated with α2-3,6,8 neuraminidase from *Clostridium perfringens,* as expected, showed more desialylation. Platelets stored at 4 °C showed more desialylation than those stored at 22 °C, which was determined as the lectin binding ratio (RCA-I) using flow cytometric analysis, although this difference did not show statistical significance. Furthermore, the lectin binding ratio increased proportionally in a time-dependent manner. On Day 5, statistical significance differences in PSL was observed both in platelets stored at 22 and 4 °C.

Third, structural changes in aged or refrigerated human platelets were observed using electron microscopy (SEM and TEM). Through SEM analysis, surface changes were observed on the platelet surface, which might be due to the result of vWF receptor aggregation, and thereby exposure of β-GlcNac, and multiple pseudopodia. Through TEM analysis, structural changes in the platelets were observed, including changes in the cell membrane and the cytoplasmic organelles.

Finally, the relationship between desialylation of platelets and TPO production was determined using HepG2 cells. HepG2 cells were co-cultured with isolated platelets, and hepatic *TPO* mRNA expression was measured at various time points and storage temperatures. It was demonstrated that hepatocytes incubated with aged or refrigerated platelets expressed higher levels of *TPO* mRNA than those incubated with fresh platelets. Similar to the results of flow cytometry analysis, refrigeration positively influenced the results of hepatic *TPO* mRNA expression, but the increase was not statistically significant. In addition, supernatant TPO levels increased proportionally in a time-dependent manner from Day 0 to Day 3.

In this study, platelets treated with sialidase (positive control) were also co-cultured with HepG2 cells. It was proven that sialidase induced desialylation of platelets as determined by flow cytometry analysis, but sialidase treated platelets did not significantly induce significant increases in the levels of hepatic *TPO* mRNA expression (data not shown). This study also conducted a serial analysis of platelets stored up to the 7 days. Surprisingly, the mean value of *TPO* mRNA expression on Day 7 decreased compared with that from Day 3 or Day 5 (data not shown). It might be that sialidase treatment and longer duration of platelet storage results in more deleterious changes, such as reduced or loss of viability and functional efficacy. We hypothesized that the use of old platelets increases the clearance and increases hepatic TPO production, so we do not need to stick to fresh platelets for transfusions. Instead, we want to prove the advantage of increasing TPO level in vivo by using aged platelets. Up to 5 days of storage duration, there were no significant damages on platelets for transfusion, but increase in TPO level was less significant than we expected. And refrigerated platelets showed structural damages and TPO level was not significantly increased. Therefore, extension of the storage duration to 7 days is difficult with the current blood processing technology. In addition, some studies defined old platelets as those with storage duration of ≥ 4 days, and demonstrated that the transfusion of these old platelets increases the risk of adverse reactions or worse outcomes [23, 24].

On the other hand, this study did not investigate the effect of storage duration and temperature on the JAK2-STAT3. This is a limitation of this study because, as mentioned above, the mechanism of these relationships is known as the result due to JAK2-STAT3 pathway [1, 19]. Therefore, further studies are required, and the risk of bacterial contamination and PSL should be assessed in human model in vivo.

This study demonstrated that aged or refrigerated platelets are more desialylated and do not show comparable structural and functional integrity with fresh platelets. And this study indicates that desialylated platelets due to aging or refrigeration are cleared more rapidly than fresh platelets by hepatic endocytic AMR, and hepatic TPO production is increased as a feedback mechanism.

Conventional platelet storage guideline is currently limited under 22 °C for 5 days. Refrigeration or cold storage may be an attractive alternative for preventing from bacterial contamination, so there have been some studies about platelet refrigeration [25–27]. However, our study indicates that both longer storage duration and refrigeration of platelets result in deleterious changes as proven in previous studies. Thus, this study suggests that current platelet storage guideline remains valid with the present technology.

## Conclusion

In conclusion, this study demonstrated that, in vitro, aging and refrigeration affect the integrity of human platelets in terms of their structural and functional aspects; this induces in the stimulation of hepatic *TPO* mRNA expression, as proved in mouse model [1]. Storage duration and refrigeration alters the integrity of platelets, thereby causing β-GlcNac exposure. Desialylated platelets are taken up by AMR on hepatocytes, and hepatic *TPO* mRNA expression is triggered. To the best of our knowledge, this is the first human study which demonstrates a relationship between desialylation of platelets and TPO production in hepatocytes according to storage temperature and duration in vitro. Furthermore, these results may help others to better study and establish more appropriate and better storage strategies and conditions for platelet components.

## Abbreviations

PC: platelet concentrate
PSL: platelet storage lesion
GP: glycoprotein
vWF: von Willebrand factor
β-GlcNac: β-*N*-acetylglucosamine
TPO: thrombopoietin
HSC: hematopoietic stem cell
Mpl: myeloproliferative leukemia oncogene
AMR: Ashwell–Morell receptor
WB: whole blood
CPDA: citrate phosphate dextrose adenine
RBC: red blood cell
IRB: institutional review board
CBC: complete blood cell
FITC: fluorescein isothiocyanate
RCA-I: *Ricinus Communis* agglutinin I
FSC: forward scatter characteristic
SSC: side scatter characteristic
SEM: scanning electron microscopy
TEM: transmission electron microscopy
PB: phosphate buffer
DMEM: Dulbecco’s modified Eagle’s medium
FBS: fetal bovine serum
P/S: penicillin/streptomycin
PCR: polymerase chain reaction
MMLC: Moloney murine leukemia virus
qPCR: quantitative PCR
ANOVA: analysis of variance
